# The Impact of Recent Developments in Electrochemical POC Sensor for Blood Sugar Care

**DOI:** 10.3389/fchem.2021.723186

**Published:** 2021-07-29

**Authors:** Wei Li, Weixiang Luo, Mengyuan Li, Liyu Chen, Liyan Chen, Hua Guan, Mengjiao Yu

**Affiliations:** ^1^ ICU of Shenzhen People’s Hospital, 2nd Clinical Medical College of Jinan University, Shenzhen, China; ^2^ Nursing Department of Shenzhen People’s Hospital, 2nd Clinical Medical College of Jinan University, Shenzhen, China; ^3^ Hepatological Surgery Department of Shenzhen People’s Hospital, 2nd Clinical Medical College of Jinan University, Shenzhen, China; ^4^ Endocrinology Department of Shenzhen People’s Hospital, 2nd Clinical Medical College of Jinan University, Shenzhen, China; ^5^ Respiratory Department of Shenzhen People’s Hospital, 2nd Clinical Medical College of Jinan University, Shenzhen, China; ^6^ Gastroenterology Department of Shenzhen People’s Hospital, 2nd Clinical Medical College of Jinan University, Shenzhen, China

**Keywords:** enzyme-free electrochemical sensor, point of care, blood sugar care, graphene, carbon materials, enzymatic electrochemical sensor

## Abstract

Rapid glucose testing is very important in the care of diabetes. Monitoring of blood glucose is the most critical indicator of disease control in diabetic patients. The invention and popularity of electrochemical sensors have made glucose detection fast and inexpensive. The first generation of glucose sensors had limitations in terms of sensitivity and selectivity. In order to overcome these problems, scientists have used a range of new materials to produce new glucose electrochemical sensors with higher sensitivity, selectivity and lower cost. A variety of different electrochemical sensors including enzymatic electrochemical sensors and enzyme-free electrochemical sensors have been extensively investigated. We discussed the development process of electrochemical glucose sensors in this review. We focused on describing the benefits of carbon materials in nanomaterials, specially graphene for sensors. In addition, we discussed the limitations of the sensors and challenges in future research.

## Introduction

Blood glucose a very important indicator in medical care due to the close relationship between the level of sugar in the blood and many diseases, such as cardiovascular disease, type II diabetes and obesity. Among them, diabetes is an important chronic disease faced by modern people (Klonoff et al., 2018; Zhu et al., 2020). It has a very high number of patients in both developed and developing countries. Since diabetes leads to metabolic disorders, there is a close link between it and many other diseases, such as kidney disease, nerve damage and heart disease (Chen et al., 2017; Pandey et al., 2017). The monitoring of blood glucose is a very important part of the diagnosis and treatment of diabetes. Therefore, how to quickly test blood glucose is a very important topic in medicine (Pleus et al., 2018).

Electrochemical sensors are the most commonly used method in glucose testing and have been successfully commercialized (Long et al., 2018; Karimi-Maleh et al., 2020; Zhang et al., 2020; Zheng et al., 2020). Electrochemical glucose sensors include enzymatic and non-enzymatic sensors. Among them, non-enzymatic electrochemical sensors are based on the direct electrochemical oxidation of glucose on the electrode surface (Hwang et al., 2018; Ding et al., 2019; Li et al., 2019; Karimi-Maleh et al., 2021). Enzymatic electrochemical sensors are based on the specific reaction between glucose oxidase and glucose to generate a detection signal (Sehit and Altintas, 2020). For both enzymatic and non-enzymatic electrochemical sensors, the use of suitable nanomaterials in the assembly process can improve the sensitivity of the sensor (Batool et al., 2019; Fan et al., 2021).

In this review, we first discussed the differences between enzymatic and non-enzymatic sensors and compare the two technologies. Then, we introduced the current state-of-the-art in carbon nanomaterials, special graphene for glucose sensors with challenges and opportunities.

## Generations of Electrochemical Glucose Sensors

The glucose sensor consists of a modified electrode that selectively catalyzes the oxidation of glucose on the electrode surface and a transducer that converts the chemical signal of the reaction into an electrical signal that is displayed by an instrument (Zhang et al., 2021). Various types of glucose sensors can be constructed by applying different modified electrodes. According to the presence of glucose oxidase (GOx) in the modified electrode, glucose sensors can be divided into two categories: GOx sensors and non-enzymatic glucose (NEG) sensors.

GOx sensors are formed by immobilizing GOx on the surface of a modified electrode in combination with an electrochemical device (Baghayeri et al., 2017; Hou et al., 2018; Mano, 2019; German et al., 2020; Suzuki et al., 2020; Lipińska et al., 2021; Wu et al., 2021). The first enzyme-based modified electrode was developed by Clark and Lyons in 1962 (Clark and Lyons, 1962). The first enzyme-based modified electrode was prepared by Updike and Hicks in 1967 as an electrochemical glucose sensor for the quantitative determination of glucose in serum (Updike and Hicks, 1967). Since then, GOx sensors have been extensively studied and different types of GOx sensors have been fabricated (Chen et al., 2013). According to the different electron acceptors, there are three generations of GOx sensors.

The first generation GOx sensor uses oxygen as the electron acceptor. GOx reduces oxygen to hydrogen peroxide in the presence of glucose and determines the glucose concentration by measuring the decrease in oxygen concentration or the increase in hydrogen peroxide concentration during the reaction (Abellán-Llobregat et al., 2017; Campbell et al., 2017; Bagdžiūnas and Palinauskas, 2020). However, the first generation sensors are susceptible to the oxygen concentration in the detection environment and have poor anti-interference property (Okuda-Shimazaki et al., 2020; Walker and Dick, 2021). At a high potential level some coexisting species such as ascorbic acid and uric acids are electroactive, reducing the selectivity and accuracy of the sensors (Choi et al., 2019; Lee et al., 2019). This problem can be minimized by using a permselective membrane, reducing the access of the interferent to the surface of the sensor transducer.

The second-generation GOx sensor uses an electron transfer mediator instead of oxygen as the electron acceptor, which can overcoming the oxygen limitation of the first-generation GOx sensor (Lin et al., 2019; Yadav et al., 2019). The electron mediators are small, soluble redox-active molecules such as ferrocene derivatives, ferricyanides, conductive organic salts and quinones. These molecules can perform rapid and reversible redox reactions. They accelerate the shuttling of electrons between the active site of the enzyme and the electrode surface, increasing the rate of enzymatic reactions (Mahajan et al., 2018). However, the electron mediator can easily diffuse out of the enzyme layer into the substrate solution, which affects the stability of the sensor.

The third generation GOx sensor does not require oxygen molecules or electron transfer mediator molecules as electron acceptors compared with the previous two generations of GOx sensors (Mehmeti et al., 2017; Çakıroğlu and Özacar, 2017; Dahiya et al., 2020). They are made by immobilizing the enzyme directly on the modified electrode, so that the active site of the enzyme is in close proximity to the electrode for direct electron transfer. This can improve the sensitivity and selectivity of the glucose sensor. The materials used to immobilize the enzyme are often organic conductive composite membranes, organic conductive polymer membranes, metallic nanoparticles or non-metallic nanoparticles (Meng et al., 2018; Xie et al., 2018; Ding et al., 2019; Li et al., 2019; Wang et al., 2019; Shahhoseini et al., 2019). However, the electron transfer rate of third generation GOx sensors is still limited. GOx sensors have good selectivity and sensitivity, but there are still some problems, such as the complex immobilization process of enzymes, which is prone to deactivation and denaturation. The amount of enzymes immobilized each time cannot be accurately controlled (Liu et al., 2017; Khalaf et al., 2020). In addition, the use of enzymes is limited by external conditions such as temperature, pH and humidity (Xu et al., 2017; Parashuram et al., 2019). Therefore, the development of enzyme-free glucose (NEG) sensors is particularly important. Moreover, the biosensor performance also depends on the enzymatic layer thickness with high layer thickness resulting in signal dampening or loss.

The modified electrode surface of NEG does not contain GOx. Depending on the electrochemical detection method, NEG electrochemical sensors can be divided into three categories: potentiometric, voltammetric, and current sensors (Yang et al., 2017; Zheng et al., 2018).

## Nobel Metal-Modified Glucose Sensor

Several metals, especially noble metals, have been studied as a base material for the electrodes of non-enzymatic glucose sensors. As a result, a deeper understanding of the glucose direct oxidation mechanism was achieved, showing that the mechanism depends directly on the metallic catalyst used in the electrode (Zhong et al., 2017; Wang et al., 2019). Moreover, advances in material science led to the development of several metal alloys and hybrid materials, allowing for improved properties when compared to noble metals and metal oxides alone.

Pt metal is one of the earliest and most widely used electrode materials in glucose sensor because of its good catalytic activity for the oxidation of many compounds, especially glucose. However, Cl^−^ and other interferences in the solution can strongly adsorb on the Pt electrode surface, occupying the active site and reducing the catalytic activity, which seriously hinders the application of Pt in glucose sensors.

Au electrode is very active in the catalytic oxidation of glucose and have good biocompatibility, making them an excellent electrode material. However, the adsorption of Au electrodes to glucose is much less than that of Pt electrodes and is also susceptible to interference by Cl^−^, which affects its catalytic activity and stability (Toghill and Compton, 2010).

The catalytic oxidation of glucose by Pd as an electrode has a high activity. In addition, Pd is cheaper than other noble metals. However, Pd nanoparticles are very prone to polymerization and the catalytic activity can only be maintained for a few minutes. In order to improve the stability of Pd, many modification methods have been investigated.

Metallic Ni electrodes have a very high sensitivity up to 36 6 mA mM/cm^2^, while Ni electrodes are not interfered with by Cl^−^ and have good stability. However, many organic small molecules can be oxidized on the Ni electrode surface, resulting in poor selectivity of the metal Ni electrode for glucose and a narrow linear range for glucose detection (Fleischmann et al., 1971). To address these drawbacks of pure Ni electrodes, scientists have investigated the application of nanostructured Ni with high specific surface area and its oxidants in NEG sensors.

Metallic Cu electrodes are easy to prepare and inexpensive, which have been widely studied and used for NEG sensors in recent years. However, the strong adsorption of Cl^−^ on the surface of Cu electrode interferes with the detection of glucose. In addition, the electrode has a narrow detection range for glucose. Meher et al. (Meher and Rao, 2013) prepared sandwich structured CuO electrodes by homogeneous deposition under the action of microwave. This electrode has a high specific surface area and pore volume, which in turn improves the sensitivity of the sensor. The response time for the detection of glucose was only 0.7 s and the detection limit was 1 μM. Meanwhile, its stability is very good, with a sensitivity loss of only 1.3% after 1 month of use. However, it has a narrow linear range of 0–3 mM.

## Carbon Materials-Modified Glucose Sensor

Carbon materials, including fullerenes, diamond, carbon nanotubes, graphene, and carbon nanofibers, have been widely used as electrode materials for NEG sensors due to their excellent electrical conductivity and electrochemical inertness. Among them, graphene is most widely used.

Graphene is a planar hexagonal lattice material formed by sp^2^-hybridized carbon atoms connected by covalent bonds. Its unique electronic structure characteristics and physicochemical properties make it show unique advantages in electrochemical detection and electroanalysis. It can be used to prepare electrochemical sensors for bioanalysis and environmental detection with high sensitivity, good selectivity, fast current response, wide detection range and low detection limit. The high electrical conductivity of graphene and the large number of boundary points, structural defects and functional groups in its structure provide rich sites for adsorption and electrochemical reactions. This can accelerate electron transfer and enable direct electrochemical reaction and biosensing. Meanwhile, compositionally and structurally rich graphene derivatives offer the possibility to further tune their electrochemical properties. Graphene with different structural features, such as graphene nanoblankets, nanosheets, flake crystals, nanofibers, nanoribbons and quantum dots, have been successively applied to electrochemical investigations.

However, single graphene cannot meet all the requirements for electrochemical detection. The curling, agglomeration, stacking between layers and its dispersion in solvent of graphene itself limit its application in electrochemistry. Therefore, it is necessary to further improve the electrochemical properties and enhance the electrochemical effects of graphene by compounding it with other functional nanomaterials such as inorganic and organic components.

There are many electrochemical sensing methods based on graphene nanocomposites, and the most commonly used ones are electrochemical impedance method/cyclic voltammetry and chrono-current method. Among them, electrochemical impedance spectroscopy is a powerful tool to study the nature of electron transfer on the electrode surface. The Nyquist plot of the impedance spectrum consists of two parts: the semicircular part in the high frequency region corresponds to the electron transfer limitation process. The linear part in the low-frequency region corresponds to the diffusion process. The electron transfer impedance is equal to the diameter of the semicircle. The electron transfer impedance can be obtained from the size of the semicircle diameter of different modified electrodes, and thus the modification of the electrode surface and the electrical properties of different electrode modification materials. Cyclic voltammetry is used to compare the differences in peak current and peak potential between electrodes with different material modifications and different test conditions. This is combined with the scan rate, pH, and concentration of the test substance to obtain visual data for electrochemical performance analysis. The relationship between current and time can be characterized by the chrono-current method when different concentrations of the test substance are added continuously to the test electrolyte. It can obtain specific experimental data such as linear detection range, detection limit and sensitivity from the chrono-current curve.

Hossain and Slaughter (2020) proposed a hybrid glucose biosensor with high sensitivity and selectivity using both multi-walled carbon nanotubes (MWCNTs) and graphene. Chemically derived graphene and MWCNTs functionalized with carboxylic groups were synthesized using a one-step solvothermal technique to produce a suspension containing both materials. This suspension was then drop casted on a Au electrode forming a thin film onto which PtNPs were electrochemically deposited. Finally, GOx was immobilised on the nanostructured electrode and coated with Nf.

Noble metal nanoparticles (NPs) have a great specific surface area, considerable electrical conductivity and reactivity. The sufficient number of surface active sites endows them with excellent electroanalytical and electrocatalytic properties. Combining them with enzymes can act as excellent wires or electron channels to accelerate the electron exchange between the electrode and the redox protein/enzyme interface. Also, as a biocompatible material, it can provide a microenvironment similar to its nature for the immobilization of biomolecules such as proteins and enzymes. This maintains their enzymatic and electrochemical activity and allows more free orientation of protein and enzyme molecules. This can reduce the insulating nature of the protein shell or enzyme 3D structure in direct electron transfer. Immobilization of NPs on the surface of the substrate electrode is a very important step in the preparation of composite electrocatalytic systems based on NPs. Graphene can be used as a conductive carrier for the deposition of electrocatalytic NPs.

Niu et al. (Shan et al., 2010) found that the electrocatalytic activity of graphene composite with AuNPs for H_2_O_2_ and O_2_ increased significantly and showed a wide linear response range, high sensitivity and good reproducibility. Li et al. (Zhou et al., 2010) and Ramaprabhu et al. (Baby et al., 2010) further adsorbed GOD on graphene-AuNP composite electrode to prepare glucose biosensor. The results confirmed the effective retention of bioactivity of GOD in graphene-AuNPs composites. Cyclic voltammograms showed a fast and sensitive response to glucose with typical catalytic oxidation and showed good reproducibility, low detection limits and long-term stability. This excellent property can be attributed to the synergistic effect of graphene with AuNPs and the biocompatibility of the composite. In addition, it can be attributed to the fact that AuNPs prevent the restacking of graphene layers, leading to an increased specific surface area and enhanced sensing performance.

PdNPs are efficient catalysts for chemical conversions such as C-C bond formation, hydrogenation, hydrodehydrogenation, carboxylation and oxidation. It shows excellent sensitivity and selectivity for glucose oxidation. Zhang et al. (Lu et al., 2011) prepared an enzyme-free electrochemical biosensor using graphene-PdNP hybrid material modified electrode and used it for glucose detection. Nafion-graphene was first assembled on the electrode and chemisorbed Pd^2+^, followed by *in situ* formation of PdNPs on the electrode by reduction of Pd^2+^ by hydrazine hydrate. In alkaline medium, this graphene-PdNP hybrid modified electrode has very high electrochemical activity for the electrocatalytic oxidation of glucose. It can quantify the glucose concentration in a wide linear range of 10 μΜ-5 mM. The experimental results also showed that the sensor has good reproducibility and long-term stability. It exhibited high selectivity without any interference in the presence of other substances. Similarly, Jiang et al. (Zeng et al., 2011) covalently functionalized graphene with chitosan (CS) to improve its biocompatibility and hydrophilicity. They then further modified PdNPs using an *in situ* reduction method. the CS maintained its structure intact on graphene and the PdNPs were densely modified on graphene without agglomeration. A novel glucose biosensor was prepared by covalently immobilizing GOD on the obtained nanocomposite coating-modified glassy carbon electrode. Due to the synergistic effect of PdNPs and graphene, the electrode showed excellent electrocatalytic activity towards H_2_O_2_ and promoted a high loading of the enzyme.

Graphene compounded with metal oxide/semiconductor NPs has gained widespread attention because of its excellent electrocatalytic, electrochemical sensing and electrochemical energy conversion properties. The large surface area and high electrical conductivity of graphene itself make it an ideal two-dimensional catalyst carrier for loading metal oxide/semiconductor NPs and provides properties such as selective catalysis or sensing.

Mao et al. (2021) investigated the use of reduced graphene oxide (rGO) to increase the sensitivity and selectivity of a zinc oxide (ZnO) nanorod based biosensor. In this case, a polyethylene terephthalate (PET) substrate was used to hydrothermally synthetise the ZnO nanorods. Then, electrodeposited rGO was used to coat the ZnO/PET working electrode and AuNPs were dispersed on the surface leading to the production of ZnO/rGO/Au/PET. Finally, the GOx was physically adsorbed on the surface of the electrode leading to the fabrication of a GOx/Rgo/ZnO/Au/PET glucose biosensor with a sensitivity of 56.32 μA mM/cm^2^ and a linear range from 0.1 to 12 mM.

Graphene can be synergistically optimized not only by nanocomposites with inorganic materials, but also by organic materials for modification or functionalization. They provide a novel and efficient platform for immobilizing redox enzymes, achieving direct electrochemistry, and applying to the design and preparation of third-generation electrochemical sensors. Nafion is a perfluorinated sulfonate ionomer cross-linked polymer. Due to the advantages of simple preparation, excellent electrical conductivity, high chemical stability and biocompatibility, it has been widely used as a protective and selective coating material as well as a carrier for enzyme immobilization in biosensors. Moreover, Nafion films are negatively charged and some foreign substances into AA, UA and *p*-vinylaminophen are easily excluded during detection. Chen et al. (2010) modified GCE after immobilization of GOD on graphene/Nafion films and used it as an electrochemiluminescence sensor for glucose detection. It was shown that GOD maintained good bioactivity after immobilization on the composite film. The sensor showed a linear response of 2–100 μM for glucose with a detection limit of 1 μM. Al-Sagur et al. (2017) synthesised a multifunctional conducting polyacrylic acid (PAA) hydrogel (MFH) integrated with reduced graphene oxide (rGO), vinyl substituted polyaniline (VS-PANI), and lutetium phthalocyanine (LuPc2) to create a three dimensional (3D) robust matrix for GOx immobilization and glucose measurement.

## Conclusion and Future Perspectives

Enzymatic-based glucose biosensors seem to correspond to the ideal model for glucose biosensing. However, many challenges such as short operational lifetime, temperature, and pH range, are limiting their performance requiring the use of more advanced materials and fabrication techniques. Contrary to enzymatic sensors, non-enzymatic glucose sensing presents higher stability, selectivity, less complex manufacturing procedures, and clinical uses.

Compounding graphene with nanomaterials is an effective way to enhance functionality, and these graphene composite based biosensors show excellent sensitivity and selectivity for glucose detection. However, the development of graphene-based materials and devices is still in its infancy, and there is a need to continue to expand the scientific research of these materials and devices in the field of electroanalysis and electrocatalysis in the future. First, novel methods should be developed for the controlled synthesis and processing of graphene. Second, because graphene in composites is highly susceptible to complex interactions with other molecules leading to agglomeration, suitable methods should also be found to control the morphology and size of other functional nanomaterials on the graphene surface. In addition, the physical and chemical properties of the graphene surface, the interactions between chemicals and biomolecules at the interface and graphene should be studied in more depth. For example, the adsorption mechanism of molecules on graphene, the orientation of biomolecules on graphene Yao the interaction between graphene and biomolecules and the mechanism of its effect on the electron transport properties of graphene These studies can provide a deeper understanding of the electrochemical properties of graphene and its composites, which can facilitate the application of graphene in glucose sensors.

## References

[B1] Abellán-Llobregat A. Jeerapan I. Bandodkar A. Vidal L. Canals A. Wang J. (2017). A Stretchable and Screen-Printed Electrochemical Sensor for Glucose Determination in Human Perspiration. Biosens. Bioelectron. 91, 885–891. 10.1016/j.bios.2017.01.058 28167366 PMC5328638

[B2] Al-Sagur H. Komathi S. Khan M. A. Gurek A. G. Hassan A. (2017). A Novel Glucose Sensor Using Lutetium Phthalocyanine as Redox Mediator in Reduced Graphene Oxide Conducting Polymer Multifunctional Hydrogel. Biosens. Bioelectron. 92, 638–645. 10.1016/j.bios.2016.10.038 27836595

[B3] Baby T. T. Aravind S. S. J. Arockiadoss T. Rakhi R. B. Ramaprabhu S. (2010). Metal Decorated Graphene Nanosheets as Immobilization Matrix for Amperometric Glucose Biosensor. Sensors Actuators B: Chem. 145, 71–77. 10.1016/j.snb.2009.11.022

[B4] Bagdžiūnas G. Palinauskas D. (2020). Poly (9H-Carbazole) as a Organic Semiconductor for Enzymatic and Non-enzymatic Glucose Sensors. Biosensors 10, 104. 10.3390/bios10090104 PMC7560144 32842552

[B5] Baghayeri M. Veisi H. Ghanei-Motlagh M. (2017). Amperometric Glucose Biosensor Based on Immobilization of Glucose Oxidase on a Magnetic Glassy Carbon Electrode Modified with a Novel Magnetic Nanocomposite. Sensors Actuators B: Chem. 249, 321–330. 10.1016/j.snb.2017.04.100

[B6] Batool R. Rhouati A. Nawaz M. H. Hayat A. Marty J. L. (2019). A Review of the Construction of Nano-Hybrids for Electrochemical Biosensing of Glucose. Biosensors 9, 46. 10.3390/bios9010046 PMC6468850 30934645

[B7] Çakıroğlu B. Özacar M. (2017). Tannic Acid Modified Electrochemical Biosensor for Glucose Sensing Based on Direct Electrochemistry. Electroanalysis 29, 2719–2726. 10.1002/elan.201700420

[B8] Campbell A. S. Islam M. F. Russell A. J. (2017). Intramolecular Electron Transfer through Poly-Ferrocenyl Glucose Oxidase Conjugates to Carbon Electrodes: 1. Sensor Sensitivity, Selectivity and Longevity. Electrochimica Acta 248, 578–584. 10.1016/j.electacta.2017.07.150

[B9] Chen C. Xie Q. Yang D. Xiao H. Fu Y. Tan Y. (2013). Recent Advances in Electrochemical Glucose Biosensors: a Review. RSC Adv. 3, 4473–4491. 10.1039/c2ra22351a

[B10] Chen X. Ye H. Wang W. Qiu B. Lin Z. Chen G. (2010). Electrochemiluminescence Biosensor for Glucose Based on Graphene/nafion/GOD Film Modified Glassy Carbon Electrode. Electroanalysis 22, 2347–2352. 10.1002/elan.201000095

[B11] Chen Y. Lu S. Zhang S. Li Y. Qu Z. Chen Y. (2017). Skin-like Biosensor System via Electrochemical Channels for Noninvasive Blood Glucose Monitoring. Sci. Adv. 3, e1701629. 10.1126/sciadv.1701629 29279864 PMC5738229

[B12] Choi Y.-B. Kim H.-S. Jeon W.-Y. Lee B.-H. Shin U. S. Kim H.-H. (2019). The Electrochemical Glucose Sensing Based on the Chitosan-Carbon Nanotube Hybrid. Biochem. Eng. J. 144, 227–234. 10.1016/j.bej.2018.10.021

[B13] Clark L. C. Jr Lyons C. (1962). Electrode Systems for Continuous Monitoring in Cardiovascular Surgery. Ann. N. Y. Acad. Sci. 102, 29–45. 10.1111/j.1749-6632.1962.tb13623.x 14021529

[B14] Dahiya A. S. Thireau J. Boudaden J. Lal S. Gulzar U. Zhang Y. (2020). Review-Energy Autonomous Wearable Sensors for Smart Healthcare: A Review. J. Electrochem. Soc. 167, 037516. 10.1149/2.0162003jes

[B15] Ding L. Yan J. Zhao Z. Li D. (2019). Synthesis of NiGa2O4 Nanosheets for Non-enzymatic Glucose Electrochemical Sensor. Sensors Actuators B: Chem. 296, 126705. 10.1016/j.snb.2019.126705

[B16] Fan B. Wang Q. Wu W. Zhou Q. Li D. Xu Z. (2021). Electrochemical Fingerprint Biosensor for Natural Indigo Dye Yielding Plants Analysis. Biosensors 11, 155. 10.3390/bios11050155 34068869 PMC8153556

[B17] Fleischmann M. Korinek K. Pletcher D. (1971). The Oxidation of Organic Compounds at a Nickel Anode in Alkaline Solution. J. Electroanalytical Chem. Interfacial Electrochemistry 31, 39–49. 10.1016/s0022-0728(71)80040-2

[B18] German N. Ramanaviciene A. Ramanavicius A. (2020). Formation and Electrochemical Evaluation of Polyaniline and Polypyrrole Nanocomposites Based on Glucose Oxidase and Gold Nanostructures. Polymers 12, 3026. 10.3390/polym12123026 PMC7766309 33348805

[B19] Hossain M. F. Slaughter G. (2020). PtNPs Decorated Chemically Derived Graphene and Carbon Nanotubes for Sensitive and Selective Glucose Biosensing. J. Electroanalytical Chem. 861, 113990. 10.1016/j.jelechem.2020.113990

[B20] Hou C. Zhao D. Wang Y. Zhang S. Li S. (2018). Preparation of Magnetic Fe3O4/PPy@ZIF-8 Nanocomposite for Glucose Oxidase Immobilization and Used as Glucose Electrochemical Biosensor. J. Electroanalytical Chem. 822, 50–56. 10.1016/j.jelechem.2018.04.067

[B21] Hwang D.-W. Lee S. Seo M. Chung T. D. (2018). Recent Advances in Electrochemical Non-enzymatic Glucose Sensors - A Review. Analytica Chim. Acta 1033, 1–34. 10.1016/j.aca.2018.05.051 30172314

[B22] Karimi-Maleh H. Ayati A. Davoodi R. Tanhaei B. Karimi F. Malekmohammadi S. (2021). Recent Advances in Using of Chitosan-Based Adsorbents for Removal of Pharmaceutical Contaminants: A Review. J. Clean. Prod. 291, 125880. 10.1016/j.jclepro.2021.125880

[B23] Karimi-Maleh H. Kumar B. G. Rajendran S. Qin J. Vadivel S. Durgalakshmi D. (2020). Tuning of Metal Oxides Photocatalytic Performance Using Ag Nanoparticles Integration. J. Mol. Liquids 314, 113588. 10.1016/j.molliq.2020.113588

[B24] Khalaf N. Ahamad T. Naushad M. Al-hokbany N. Al-Saeedi S. I. Almotairi S. (2020). Chitosan Polymer Complex Derived Nanocomposite (AgNPs/NSC) for Electrochemical Non-enzymatic Glucose Sensor. Int. J. Biol. Macromolecules 146, 763–772. 10.1016/j.ijbiomac.2019.11.193 31778696

[B25] Klonoff D. C. Parkes J. L. Kovatchev B. P. Kerr D. Bevier W. C. Brazg R. L. (2018). Investigation of the Accuracy of 18 Marketed Blood Glucose Monitors. Dia Care 41, 1681–1688. 10.2337/dc17-1960 29898901

[B26] Lee I. Loew N. Tsugawa W. Ikebukuro K. Sode K. (2019). Development of a Third-Generation Glucose Sensor Based on the Open Circuit Potential for Continuous Glucose Monitoring. Biosens. Bioelectron. 124-125, 216–223. 10.1016/j.bios.2018.09.099 30388564

[B27] Li Y. Xie M. Zhang X. Liu Q. Lin D. Xu C. (2019). Co-MOF Nanosheet Array: a High-Performance Electrochemical Sensor for Non-enzymatic Glucose Detection. Sensors Actuators B: Chem. 278, 126–132. 10.1016/j.snb.2018.09.076

[B28] Lin M.-J. Wu C.-C. Chang K.-S. (2019). Effect of Poly-L-Lysine Polycation on the Glucose Oxidase/Ferricyanide Composite-Based Second-Generation Blood Glucose Sensors. Sensors 19, 1448. 10.3390/s19061448 PMC6470914 30934546

[B29] Lipińska W. Siuzdak K. Karczewski J. Dołęga A. Grochowska K. (2021). Electrochemical Glucose Sensor Based on the Glucose Oxidase Entrapped in Chitosan Immobilized onto Laser-Processed Au-Ti Electrode. Sens. Actuators B Chem. 330, 129409. 10.1016/j.snb.2020.129409

[B30] Liu G.-q. Zhong H. Li X.-r. Yang K. Jia F.-f. Cheng Z.-p. (2017). Research on Nonenzymatic Electrochemical Sensor Using HO-BiONO3 Nanocomposites for Glucose Detection. Sensors Actuators B: Chem. 242, 484–491. 10.1016/j.snb.2016.11.019

[B31] Long W. Xie Y. Shi H. Ying J. Yang J. Huang Y. (2018). Preparation of Nitrogen-Doped Hollow Carbon Spheres for Sensitive Catechol Electrochemical Sensing. Fullerenes, Nanotubes and Carbon Nanostructures 26, 856–862. 10.1080/1536383x.2018.1512973

[B32] Lu L.-M. Li H.-B. Qu F. Zhang X.-B. Shen G.-L. Yu R.-Q. (2011). *In Situ* synthesis of Palladium Nanoparticle-Graphene Nanohybrids and Their Application in Nonenzymatic Glucose Biosensors. Biosens. Bioelectron. 26, 3500–3504. 10.1016/j.bios.2011.01.033 21342759

[B33] Mahajan A. Banik S. Chowdhury S. R. Roy P. S. Bhattacharya S. K. (2018). Size Control Synthesis and Amperometric Sensing Activity of Palladium Nanoparticles for Glucose Detection. Mater. Today Proc. 5, 2049–2055. 10.1016/j.matpr.2017.09.200

[B34] Mano N. (2019). Engineering Glucose Oxidase for Bioelectrochemical Applications. Bioelectrochemistry 128, 218–240. 10.1016/j.bioelechem.2019.04.015 31030174

[B35] Mao Q. Jing W. Zhou F. Liu S. Gao W. Wei Z. (2021). Depositing Reduced Graphene Oxide on ZnO Nanorods to Improve the Performance of Enzymatic Glucose Sensors. Mater. Sci. Semiconductor Process. 121, 105391. 10.1016/j.mssp.2020.105391

[B36] Meher S. K. Rao G. R. (2013). Archetypal sandwich-structured CuO for High Performance Non-enzymatic Sensing of Glucose. Nanoscale 5, 2089–2099. 10.1039/c2nr33264g 23381131

[B37] Mehmeti E. Stanković D. M. Chaiyo S. Zavasnik J. Žagar K. Kalcher K. (2017). Wiring of Glucose Oxidase with Graphene Nanoribbons: an Electrochemical Third Generation Glucose Biosensor. Microchim. Acta 184, 1127–1134. 10.1007/s00604-017-2115-5

[B38] Meng W. Wen Y. Dai L. He Z. Wang L. (2018). A Novel Electrochemical Sensor for Glucose Detection Based on Ag@ZIF-67 Nanocomposite. Sensors Actuators B: Chem. 260, 852–860. 10.1016/j.snb.2018.01.109

[B39] Okuda-Shimazaki J. Yoshida H. Sode K. (2020). FAD Dependent Glucose Dehydrogenases - Discovery and Engineering of Representative Glucose Sensing Enzymes -. Bioelectrochemistry 132, 107414. 10.1016/j.bioelechem.2019.107414 31838457

[B40] Pandey R. Paidi S. K. Valdez T. A. Zhang C. Spegazzini N. Dasari R. R. (2017). Noninvasive Monitoring of Blood Glucose with Raman Spectroscopy. Acc. Chem. Res. 50, 264–272. 10.1021/acs.accounts.6b00472 28071894 PMC5896772

[B41] Parashuram L. Sreenivasa S. Akshatha S. Udayakumar V. Sandeep kumar S. (2019). A Non-enzymatic Electrochemical Sensor Based on ZrO2: Cu(I) Nanosphere Modified Carbon Paste Electrode for Electro-Catalytic Oxidative Detection of Glucose in Raw Citrus Aurantium Var. Sinensis. Food Chem. 300, 125178. 10.1016/j.foodchem.2019.125178 31326677

[B42] Pleus S. Heinemann L. Freckmann G. (2018). Blood Glucose Monitoring Data Should Be Reported in Detail when Studies about Efficacy of Continuous Glucose Monitoring Systems Are Published. J. Diabetes Sci. Technol. 12, 1061–1063. 10.1177/1932296817753629 29322825 PMC6134630

[B43] Sehit E. Altintas Z. (2020). Significance of Nanomaterials in Electrochemical Glucose Sensors: An Updated Review (2016-2020). Biosens. Bioelectron. 159, 112165. 10.1016/j.bios.2020.112165 32291248

[B44] Shahhoseini L. Mohammadi R. Ghanbari B. Shahrokhian S. (2019). Ni(II) 1D-Coordination polymer/C60-Modified Glassy Carbon Electrode as a Highly Sensitive Non-enzymatic Glucose Electrochemical Sensor. Appl. Surf. Sci. 478, 361–372. 10.1016/j.apsusc.2019.01.240

[B45] Shan C. Yang H. Han D. Zhang Q. Ivaska A. Niu L. (2010). Graphene/AuNPs/chitosan Nanocomposites Film for Glucose Biosensing. Biosens. Bioelectron. 25, 1070–1074. 10.1016/j.bios.2009.09.024 19883999

[B46] Suzuki N. Lee J. Loew N. Takahashi-Inose Y. Okuda-Shimazaki J. Kojima K. (2020). Engineered Glucose Oxidase Capable of Quasi-Direct Electron Transfer after a Quick-And-Easy Modification with a Mediator. Int. J. mol. Sci. 21, 1137. 10.3390/ijms21031137 PMC7036908 32046321

[B47] Toghill K. E. Compton R. G. (2010). Electrochemical Non-enzymatic Glucose Sensors: a Perspective and an Evaluation. Int. J. Electrochem. Sci. 5, 1246–1301.

[B48] Updike S. J. Hicks G. P. (1967). The Enzyme Electrode. Nature 214, 986–988. 10.1038/214986a0 6055414

[B49] Walker N. L. Dick J. E. (2021). Oxidase-loaded Hydrogels for Versatile Potentiometric Metabolite Sensing. Biosens. Bioelectron. 178, 112997. 10.1016/j.bios.2021.112997 33535157 PMC7919600

[B50] Wang L. Xu M. Xie Y. Qian C. Ma W. Wang L. (2019a). Ratiometric Electrochemical Glucose Sensor Based on Electroactive Schiff Base Polymers. Sensors Actuators B: Chem. 285, 264–270. 10.1016/j.snb.2019.01.061

[B51] Wang Y. Wang X. Lu W. Yuan Q. Zheng Y. Yao B. (2019b). A Thin Film Polyethylene Terephthalate (PET) Electrochemical Sensor for Detection of Glucose in Sweat. Talanta 198, 86–92. 10.1016/j.talanta.2019.01.104 30876607

[B52] Wu M. Zhang Q. Fang Y. Deng C. Zhou F. Zhang Y. (2021). Polylysine-modified MXene Nanosheets with Highly Loaded Glucose Oxidase as cascade Nanoreactor for Glucose Decomposition and Electrochemical Sensing. J. Colloid Interf. Sci. 586, 20–29. 10.1016/j.jcis.2020.10.065 33153715

[B53] Xie F. Cao X. Qu F. Asiri A. M. Sun X. (2018). Cobalt Nitride Nanowire Array as an Efficient Electrochemical Sensor for Glucose and H2O2 Detection. Sensors Actuators B: Chem. 255, 1254–1261. 10.1016/j.snb.2017.08.098

[B54] Xu M. Song Y. Ye Y. Gong C. Shen Y. Wang L. (2017). A Novel Flexible Electrochemical Glucose Sensor Based on Gold Nanoparticles/polyaniline Arrays/carbon Cloth Electrode. Sensors Actuators B: Chem. 252, 1187–1193. 10.1016/j.snb.2017.07.147

[B55] Yadav V. D. Krishnan R. A. Jain R. Dandekar P. (2019). *In-situ* Silver Nanoparticles Formation as a Tool for Non-enzymatic Glucose Sensing: Study with an Enzyme Mimicking Salt. Colloids Surf. A: Physicochemical Eng. Aspects 580, 123715. 10.1016/j.colsurfa.2019.123715

[B56] Yang H. Wang Z. Li C. Xu C. (2017). Nanoporous PdCu alloy as an Excellent Electrochemical Sensor for H2O2 and Glucose Detection. J. Colloid Interf. Sci. 491, 321–328. 10.1016/j.jcis.2016.12.041 28049057

[B57] Zeng Q. Cheng J.-S. Liu X.-F. Bai H.-T. Jiang J.-H. (2011). Palladium Nanoparticle/chitosan-Grafted Graphene Nanocomposites for Construction of a Glucose Biosensor. Biosens. Bioelectron. 26, 3456–3463. 10.1016/j.bios.2011.01.024 21324668

[B58] Zhang L. Ren Z. Su Z. Liu Y. Yang T. Cao M. (2021). Novel Recurrent Altered Genes in Chinese Patients with Anaplastic Thyroid Cancer. J. Clin. Endocrinol. Metab. 106, 988–998. 10.1210/clinem/dgab014 33428730

[B59] Zhang M. Pan B. Wang Y. Du X. Fu L. Zheng Y. (2020). Recording the Electrochemical Profile of Pueraria Leaves for Polyphyly Analysis. ChemistrySelect 5, 5035–5040. 10.1002/slct.202001100

[B60] Zheng W. Wu H. Jiang Y. Xu J. Li X. Zhang W. (2018). A Molecularly-Imprinted-Electrochemical-Sensor Modified with Nano-Carbon-Dots with High Sensitivity and Selectivity for Rapid Determination of Glucose. Anal. Biochem. 555, 42–49. 10.1016/j.ab.2018.06.004 29908860

[B61] Zheng Y. Zhu J. Fu L. Liu Q. (2020). Phylogenetic Investigation of Yellow Camellias Based on Electrochemical Voltammetric Fingerprints. Int. J. Electrochem. Sci. 15, 9622–9630. 10.20964/2020.10.54

[B62] Zhong S.-L. Zhuang J. Yang D.-P. Tang D. (2017). Eggshell Membrane-Templated Synthesis of 3D Hierarchical Porous Au Networks for Electrochemical Nonenzymatic Glucose Sensor. Biosens. Bioelectron. 96, 26–32. 10.1016/j.bios.2017.04.038 28458131

[B63] Zhou K. Zhu Y. Yang X. Li C. (2010). Electrocatalytic Oxidation of Glucose by the Glucose Oxidase Immobilized in Graphene-Au-Nafion Biocomposite. Electroanalysis 22, 259–264. 10.1002/elan.200900321

[B64] Zhu L. She Z.-G. Cheng X. Qin J.-J. Zhang X.-J. Cai J. (2020). Association of Blood Glucose Control and Outcomes in Patients with COVID-19 and Pre-existing Type 2 Diabetes. Cel Metab. 31, 1068–1077. 10.1016/j.cmet.2020.04.021 PMC7252168 32369736

